# Optimizing Plant Density and Row Spacing Enhances Growth, Yield and Quality of Waxy Maize on the Loess Plateau

**DOI:** 10.3390/plants14182902

**Published:** 2025-09-18

**Authors:** Lin Xie, Bao-Jie Su, Ya-Nan Zhang, Dong-Sheng Zhang, Jing-Jing Han, Hui-Ming Li, Wan-Jun Feng, Tian-Qing Du, Fu-Zhu Cui, Jian-Fu Xue

**Affiliations:** 1 Key Laboratory of Sustainable Dryland Agriculture of Shanxi Province, College of Agriculture, Shanxi Agricultural University, Taiyuan 030031, China; 20232197@stu.sxau.edu.cn (L.X.);; 2 College of Agricultural Economics and Management, Shanxi Agricultural University, Jinzhong 030801, China; 3 Sorghum Research Institute, Shanxi Agricultural University, Jinzhong 030600, China

**Keywords:** plant densification, row spacing configuration, yield components, nutritional quality, purple waxy maize

## Abstract

Waxy maize (*Zea mays* L. *ceratina*) is extensively cultivated and exhibits substantial market demand in China; however, its yield and quality improvement remain constrained by relatively underdeveloped cultivation techniques. Optimizing plant density and row spacing is critical to improving the yield and nutritional quality of waxy maize, yet their combined effects remain insufficiently explored. A split-plot design evaluated two plant densities, i.e., 5.25 × 10^4^ plants ha^−1^ (PD_5.25_) and 6.75 × 10^4^ plants ha^−1^ (PD_6.75_), and three row configurations, i.e., 80 + 40 cm wide–narrow rows (RS_8-4_), 100 + 20 cm wide–narrow rows (RS_10-2_) and conventional 60 + 60 cm equal rows (RS_6-6_). This study aims to identify the optimal cultivation configuration for waxy maize in the Loess Plateau region. Results showed that the RS_8-4_ configuration maximized agronomic traits, dry matter accumulation, and yield relative to RS_6-6_ and RS_10-2_ treatments. Specifically, RS_8-4_ reduced the insertion angle of the lower ear leaf by 12.4% (*p* < 0.05) and ear height by 8.3% while increasing yield by 19.86–20.00% compared to RS_6-6_ and RS_10-2_ treatments. At fresh-market maturity, dry matter accumulation under RS_8-4_ treatment increased significantly by 34.0% with higher plant density. Under PD_6.75_, RS_8-4_ boosted dry matter by 29.8% and 39.4% versus RS_6-6_ and RS_10-2_, respectively. Under the RS_8-4_ and PD_6.5_ configurations, dry matter accumulation reached 13.56 t ha^−1^ and a yield of 9.94 t ha^−1^ was achieved in 2022. In summary, the combination of the PD_6.75_ density and the RS_8-4_ row spacing configuration achieved the optimal yield for the ‘Jinnuo 20’ cultivar in the Loess Plateau region. This approach provides a scalable planting framework for high-yield waxy maize production in the area, while demonstrating that optimized plant density and row spacing represent not only a key technical measure for enhancing productivity but also a core agronomic strategy for improving resource-use efficiency.

## 1. Introduction

Purple waxy maize (*Zea mays* L. var. *ceratina*) constitutes a specialized maize variant characterized by elevated amylopectin purity and enhanced vitamin profiles relative to conventional maize [1,2]. Due to its distinctive sensory attributes, superior nutritional composition, and economic significance, purple waxy maize has gained global commercial prominence in recent years [3,4]. Optimization of agronomic management strategies, e.g., particularly planting density and row configuration, represents a critical pathway for augmenting both yield potential and quality parameters for waxy maize. However, the characteristic wide stem–leaf angle of waxy maize often necessitates a relatively low planting density, which restricts the number of productive ears per unit area and consequently diminishes land use efficiency [5]. Although previous studies have examined the effects of planting density and row spacing in maize, most have focused on their individual impacts on yield or spatial structure, and a comprehensive understanding of their synergistic effects on yield formation, canopy architecture, and quality traits remains limited [6]. Existing studies have primarily focused on ordinary maize or general waxy maize, with limited attention given to purple waxy maize. In particular, the mechanisms through which density and row spacing configurations modulate the balance between yield and nutritional quality in purple waxy maize—through effects on light distribution and assimilate partitioning—are not yet fully understood [7]. Consequently, elucidating the synergistic effects of density–spatial arrangements on yield architecture, quality determinants, and their drivers emerges as a research imperative for purple waxy maize production systems.

Planting density represents a critical agronomic lever for optimizing crop growth dynamics, yield architecture, and quality parameters [8]. Optimizing plant density enhances crop yield by improving agronomic traits, such as plant height (*PH*), ear height coefficient (*EHC*) and leaf area distribution, along with dry matter accumulation and partitioning into grains [9]. Currently, waxy maize is typically planted at approximately 52,500 plants ha^−1^ under equidistant row spacing. However, most waxy maize varieties exhibit an open canopy architecture, limiting achievable densities under this system due to constraints such as light interception and airflow. Wide–narrow row planting offers a potential solution to increase planting density, thereby enhancing waxy maize productivity and profitability. Therefore, optimizing row spacing to facilitate higher planting densities is crucial for maximizing yields and economic returns in waxy maize cultivation.

Optimized row spacing can mitigate the negative effects of crowding stress on waxy maize growth and yield at higher planting densities. Studies demonstrate that wide–narrow row planting significantly enhances yield in high-density waxy maize cultivation [10,11]. Compared with equidistant row spacing, this layout improves plant architecture in grain maize [12], while increasing the relative chlorophyll content, photosynthetic rate [13], light energy absorption and utilization [14], and dry matter accumulation, translocation, and yield formation [15]. To our knowledge, limited information exists regarding the effects of row spacing and planting density on yield, quality and their underlying drivers in purple waxy maize.

Waxy maize, a fresh-consumption specialty crop, requires yield and nutritional quality improvements to enhance its food and economic value [16]. Typically, its nutritional quality declines as yield increases. Studies demonstrate that key traits, such as ear length and ear weight, grain protein and fat content, decrease significantly with rising plant density in waxy maize [17]. Conversely, grain starch, oil content, and other quality parameters increase under higher planting densities. This negative yield–quality trade-off is likely to be more complex in purple waxy maize due to its rich content of functional compounds. By comparison, studies on other specialty crops such as purple sweet potato and black wheat have demonstrated that optimized cultivation practices can mitigate this yield–quality trade-off, offering valuable insights for research on purple waxy maize [18]. While elevated plant density affects grain nutritional quality, such effects may be mitigated through cultivation strategies like optimized row spacing. However, whether row spacing configurations under high-density planting positively influence waxy maize nutritional quality remains unverified. Therefore, comprehensive assessment of plant density and row spacing configurations on waxy maize nutritional quality is essential.

Waxy maize is extensively cultivated across China, with a planting area exceeding 1.34 million hectares, positioning the country as the global leader in fresh maize production and consumption [19]. Despite growing market demand and continuous release of new varieties, corresponding cultivation techniques remain underdeveloped, limiting the full realization of production benefits. Moreover, factors such as the unique topography, soil types, and climatic conditions of the Loess Plateau region further constrain maize yield improvement [20]. This study is the first to systematically evaluate the interaction effects of planting density and row spacing configuration on yield formation and nutritional quality of purple waxy maize in the Loess Plateau region, aiming to (1) investigate the effect of planting density and row spacing on nutritional quality content, yield and yield components in waxy maize; (2) determine the influence of plant density and row spacing on dry matter accumulation and translocation; and (3) identify optimal cultivation practices for waxy maize production on the Loess Plateau.

## 2. Materials and Methods

### 2.1. Experimental Site

**Figure 1:**
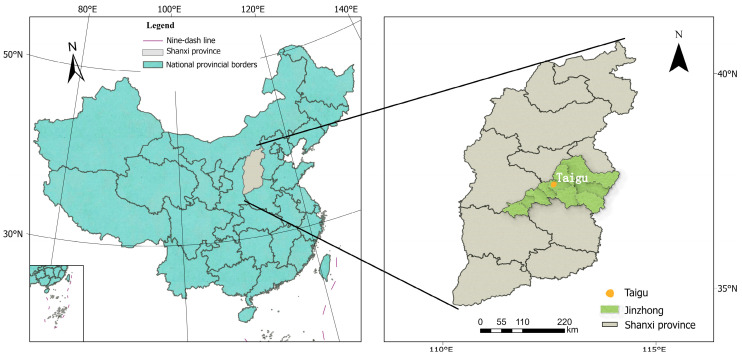
Location of the experimental site.

**Figure 2:**
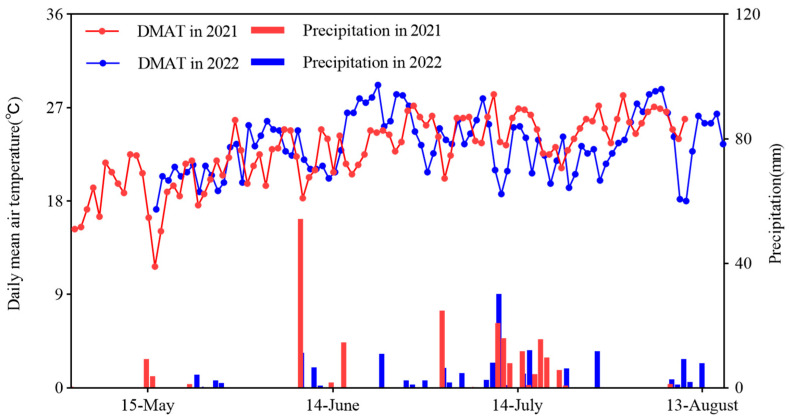
Daily mean air temperature (DMAT) and precipitation during waxy maize growing seasons in 2021 and 2022.

A field experiment was conducted in 2021 and 2022 at the Mengjiazhuang Experimental station of Shanxi Agricultural University (37°25′51″ N, 112°35′42″ E), located in Taigu country, Jinzhong City, Shanxi province (Figure 1). This region experiences a temperate continental climate, with mean annual values of 10.4 °C in temperature and 397.1 mm precipitation, a 179-day frost-free period, and a 2527.5 h sunshine duration. The experimental site features calcic cinnamon soil as the predominant soil type. Prior to treatment application, soil samples (0–20 cm depth) were collected and analyzed [21], revealing the following properties: 28.89 g kg^−1^ of soil organic matter, 1.76 g kg^−1^ of total nitrogen, 42.26 mg kg^−1^ of alkali-hydrolysable nitrogen, 15.04 mg kg^−1^ of available phosphorus, 205.69 mg kg^−1^ of available potassium, and a pH of 7.8. Total precipitation during waxy maize growing seasons reached 207.5 mm (2021) and 157.5 mm (2022), respectively (Figure 2). Daily mean air temperature throughout the growing seasons is presented in Figure 2.

### 2.2. Experimental Design

The experiment employed a split-plot design with three replications. Plant density served as the main plot factor, comprising two levels of 5.25 × 10^4^ plants ha^−1^ (PD_5.25_) and 6.75 × 10^4^ plants ha^−1^ (PD_6.75_). Row spacing configurations constituted the sub-plot factor with three treatments of wide–narrow row spacing with 80 cm + 40 cm (RS_8-4_), wide–narrow row spacing with 100 cm + 20 cm (RS_10-2_) and uniform row spacing with 60 cm + 60 cm (RS_6-6_). Individual plot dimensions measured 48 m^2^ (8 m × 6 m). All treatments received a basal application of compound fertilizer (N:P_2_O_5_:K_2_O = 18:18:18) broadcast manually before sowing, at a rate of 600 kg ha^−1^, equivalent to 108 kg N ha^−1^, 108 kg P_2_O_5_ ha^−1^ and 108 kg K_2_O ha^−1^. No topdressing was applied during the growing season. Irrigation practices followed local customary procedures. Simultaneously with irrigation, granular soil insecticides were applied manually. This was followed by rotary tillage and hand sowing. Manual weeding was performed during the seedling stage of the waxy maize. Additionally, insecticides were sprayed at both the seedling and silking stages to control insect pests. The purple waxy maize cultivar Jinnuo 20 (JN20, Maize Research Institute, Shanxi Agricultural University) was used. This cultivar exhibits a semi-compact plant architecture, with plant heights ranging from 225 to 279 cm and ear heights between 86 and 127 cm. In northern China, the growth period from emergence to fresh-ear harvest is 91.7 days. The maize was hand-sown on 16 May in both 2021 and 2022 and harvested on 10 August 2021 and 16 August 2022, respectively.

### 2.3. Sampling and Measurements

#### 2.3.1. Agronomic Traits

During the 2021–2022 growing seasons, when the third leaf of the waxy maize was fully expanded, three uniformly growing plants were randomly selected and labeled for each treatment. Agronomic traits were measured at both the seedling and silking stages. Plant height (PH) and ear height (EH) were measured using a 5-m tape measure; stem diameter (SD) was determined with digital calipers at the basal internode; the insertion angle of the primary ear leaf was measured using a digital goniometer. The measurement criteria for each indicator were as follows:

Plant height and ear height: The vertical height from the ground to the highest point of the plant and the bearing position of the top ear was measured, respectively.

Stem diameter: The diameter at the widest and narrowest points of the base of the stem was measured, and the average value was taken.

Insertion angle of the primary ear leaf: The upper angle between the stem and the leaf vein was measured.

The ear height coefficient (EHC) was calculated using Equation (1).

(1) $$\mathrm{EHC}=\frac{\mathrm{EH}}{\mathrm{PH}}$$

where EHC is the ear height coefficient, EH is the ear height (cm), and PH is the plant height (cm).

#### 2.3.2. Leaf Area Index and Leaf Area Duration

At the silking (reproductive stage 1, R1) and fresh-market maturity stage (FH), three uniformly growing plants per plot were selected for measurement of all functional leaves. Leaf length and width were recorded, with leaf area (LA, cm^2^) calculated using Equation (2).

(2) LA=l×w×0.75

where l is leaf length (cm), w is leaf width (cm), respectively, and 0.75 is the maize-specific leaf area coefficient.

Green leaf area index (LAI) was calculated using Equation (3).

(3) $$\mathrm{LAI}=\mathrm{Toal}\;\mathrm{LA}\;\mathrm{per}\;\mathrm{plant}\times \frac{n}{s}$$

where n is the number of plants per unit area (plants m^−2^), and s is land area (m^2^).

Leaf area duration (LAD) was calculated using Equations (4) and (5) [22].

(4) $${\mathrm{LAD}}_{\mathrm{ER}}=\frac{\left({\mathrm{LA}}_{E}+{\mathrm{LA}}_{R1}\right)}{2}\times \left(t_{R1}-t_{E}\right)$$

(5) $${\mathrm{LAD}}_{R}=\frac{\left({\mathrm{LA}}_{R1}+{\mathrm{LA}}_{\mathrm{FM}}\right)}{2}\times \left(t_{\mathrm{FM}}-t_{R1}\right)$$

where LAD_ER_ and LAD_R_ indicate the leaf area duration during the emergence to silking stage and the silking to fresh-eating harvest stage, respectively (m^2^ d). LA_E_, LA_R1_ and LA_FM_ indicate the leaf areas per plant at emergence, silking and fresh-market maturity stages, respectively (m^2^). t_E_, t_R1_ and t_FM_ indicate the date at emergence, silking and fresh-market maturity stages, respectively.

#### 2.3.3. Dry Matter Accumulation, Translocation and Distribution

At the silking stage, plant components were separated into vegetative organs (leaves, stems, and sheaths) and ears. At fresh-market maturity, plants were dissected into vegetative organs (leaves, stems, and sheaths), grains, and reproductive structures (bracts and cobs). All samples were oven-dried at 85 °C until a constant weight was achieved, followed by individual weighing using an electronic balance.

Dry matter translocation efficiency and its contribution to grain yield during the silking to fresh-eating harvest stage transition were quantified using Equations (6)–(8).

(6) $$\mathrm{DMT}={\mathrm{DMA}}_{\mathrm{vos}}-{\mathrm{DMA}}_{\mathrm{vom}}$$

where DMT indicates dry matter translocation (t ha^−1^), DMA_vos_ indicates dry matter accumulation in vegetative organs at the silking stage (t ha^−1^), and DMA_vom_ indicates dry matter accumulation in vegetative organs at fresh-market maturity (t ha^−1^).

(7) $$\mathrm{DMTE}=\frac{\mathrm{DMT}}{{\mathrm{MDA}}_{\mathrm{ts}}}$$

where DMTE indicates dry matter translocation efficiency (%), and DMA_ts_ indicates total plant dry matter accumulation at the silking stage (t ha^−1^).

(8) $$\mathrm{CDMG}=\frac{\mathrm{DMT}}{{\mathrm{DMA}}_{\mathrm{gm}}}$$

where CDMG indicates the proportional contribution of dry matter translocation to grain yield (%), and DMA_gm_ indicates grain dry matter accumulation at fresh-market maturity (t ha^−1^).

The harvest index (HI) was calculated using Equation (9).

(9) $$\mathrm{HI}=\frac{{\mathrm{DMA}}_{\mathrm{gm}}}{{\mathrm{DMA}}_{\mathrm{tm}}}$$

where HI indicates the harvest index, DMA_gm_ indicates the grain dry matter at fresh-market maturity (t ha^−1^), and DMA_tm_ indicates total aboveground dry matter at fresh-market maturity (t ha^−1^).

#### 2.3.4. The Yield and Its Components

At fresh-market maturity, unsampled waxy maize plants with uniform growth were harvested from a 6 m^2^ area per plot for yield determination. Ten randomly selected ears per plot were used for measurements including ear length, ear weight, ear diameter, kernel number per ear, kernel weight per ear, and 100-kernel weight.

Kernels were oven-dried at 50 °C, ground, and sieved through a 0.25-mm mesh prior to analysis. The fat content was determined using the petroleum ether-Soxhlet extraction method [23]. The reducing sugar content was determined using the 3,5-dinitrosalicylic acid colorimetric method [24]. The soluble sugar and sucrose contents were determined using the anthrone colorimetric method [25]. And the vitamin C content was determined using the molybdenum blue colorimetric method [26].

### 2.4. Statistical Analysis

Data were analyzed using SPSS 16.0 (IBM Inc., Armonk, NY, USA). The assumptions of normality and homogeneity of variances were verified for all treatments prior to conducting analysis of variance (ANOVA). Treatment means were compared via Duncan’s multiple range test at *p* < 0.05. Figures were generated in origin 2025 pro (Learning Edition, OriginLab Corp., Northampton, MA, USA).

## 3. Results

### 3.1. Agronomic Traits and Leaf Angle

**Figure 3:**
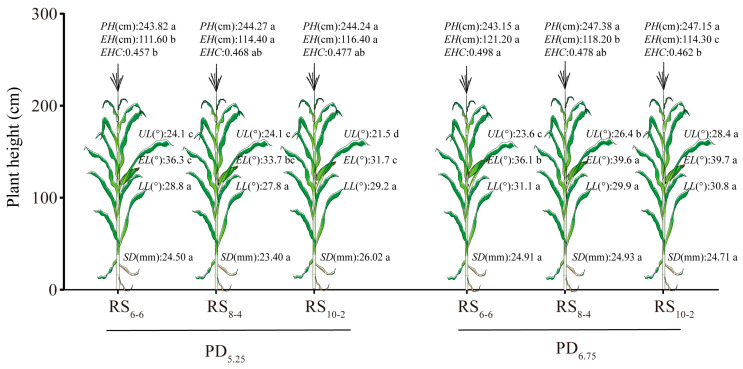
Agronomic traits and leaf insertion angle of waxy maize under different plant densities and row spacing configurations. PH, plant height. EH, ear height. EHC, ear height coefficient. UL, insertion angle of upper ear leaf. EL, insertion angle of primary ear leaf. LL, insertion angle of lower ear leaf. SD, stem diameter. Different lowercase letters indicate significant differences among treatments at *p* < 0.05.

Plant density and row spacing had no significant effect on the PH, or SD, of waxy maize (Table 1). The EH and EHC decreased with increasing row spacing under high plant density (PD_6.75_). Conversely, these parameters exhibited slight increases under low plant density (PD_5.25_). The *EHC* in the PD_6.75_ treatments was 2.59% higher than in the PD_5.25_ treatments (*p* < 0.05). The insertion angle of the upper ear leaf (UL) and primary ear leaf (EL) in PD_6.75_ treatments increased by 12.02% and 13.57%, respectively, relative to PD_5.25_ treatments (*p* < 0.05, Figure 3).

### 3.2. Leaf Area Index and Leaf Area Duration

**Figure 4:**
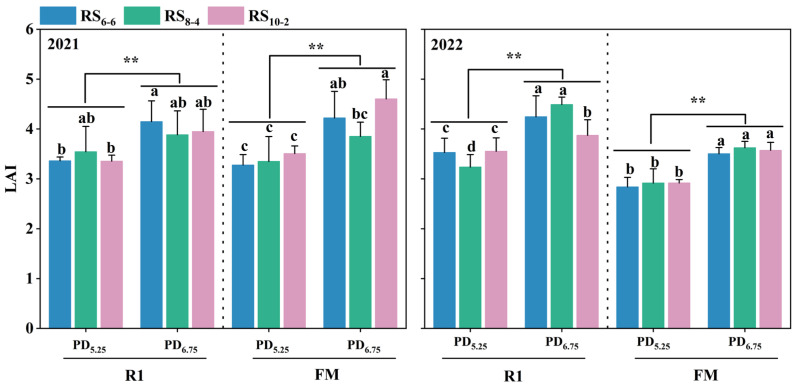
The LAI under different plant densities and row spacing treatments at the silking and fresh-market maturity stages. LAI, Leaf Area Index. R1, Reproductive stage 1. FM, Fresh market maturity. Different lowercase letters indicate significant difference among treatments at *p* < 0.05. ** indicates significant differences between PD_5.25_ and PD_6.75_ treatments at *p* < 0.01.

The LAI of waxy maize was significantly affected by plant density at both the silking and fresh-market maturity stages (Table 1). Compared with the PD_5.25_ treatment, LAI in the PD_6.75_ treatments increased by 16.67–22.51% at the silking stage and by 23.18–25.22% at fresh-market maturity (Figure 4).

**Figure 5:**
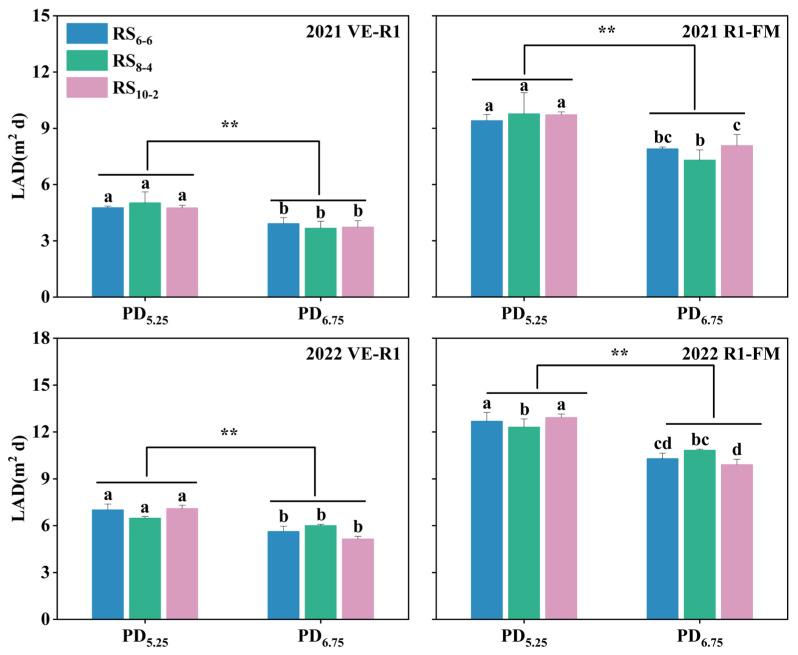
The LAD under different plant densities and row spacing treatments during emergence to silking (VE–R1) and the silking to fresh-market maturity (R1–FM) stage. LAD, Leaf Area Duration. VE, Vegetative emergence. R1, Reproductive stage 1. FM, Fresh-market maturity. Different lowercase letters indicate significant difference among treatments at *p* < 0.05. ** indicates significant differences between PD_5.25_ and PD_6.75_ treatments at *p* < 0.01.

The LAD of waxy maize decreased significantly with increasing plant density (Figure 5). Compared to the PD_5.25_ treatments, the mean LAD_ER_ in the PD_6.75_ treatments showed reductions of 19.41% and 22.21% in 2021 and 2022, respectively. Similarly, the mean LAD_R_ decreased by 18.17% and 18.54% under the same density comparison for the two consecutive years.

### 3.3. Dry Matter Accumulation

At silking stage, the DMA_vos_, DMA_es_ and DMA_ts_ increased with wider row spacing under high plant density (PD_6.75_), though no significant differences were observed among row spacing treatments (Table 2). At fresh-market maturity, the DMA_gm_ and DMA_tm_ in PD_6.75_ treatments increased significantly by 54.45% and 18.09%; under the PD_6.75_ planting density, the total dry matter weight under the RS_8-4_ row spacing configuration reached 13.56 t ha^−1^, significantly exceeding that of other treatments. Neither plant density nor row spacing significantly affected DMT, DMTE and CDMG. Moreover, the HI increased by 30.81% in PD_6.75_ treatments compared to PD_5.25_.

### 3.4. Yield and Its Components

Plant density affected the number of effective ears (NEE), kernel number per ear (KNE) and 100-kernel weight (HKW) of waxy maize (Table 3). Both KNE and HKW were significantly decreased with increasing plant density. Under the PD_6.75_ treatment in 2022, the theoretical yield (ThY) ranking among treatments was RS_8-4_ > RS_6-6_ > RS_10-2_, with the RS_8-4_ configuration exhibiting a significant increase of 20% compared to the RS_10-2_ treatment (*p* < 0.05). Under the same treatment conditions in the same year, FEY also demonstrated a consistent variation trend, although the differences among treatments were not statistically significant.

### 3.5. Ear Traits

Plant density and row spacing significantly affected ear length, weight, and diameter of waxy maize (Table 4). These three parameters decreased significantly with increasing plant density. At equivalent row spacing, ears in PD_5.25_ treatments exhibited lengths significantly greater by 5.8–19.6% in 2021 and by 4.8–12.9% in 2022 compared to PD_6.75_ treatments. Under high-density conditions (PD_6.75_), RS_8-4_ configurations yielded 10.6% and 9.4% heavier ears than RS_6-6_ and RS_10-2_ in 2021 (*p* < 0.05). In 2022, RS_6-6_ and RS_8-4_ treatments under PD_6.75_ significantly increased ear length by 10.7% and 10.6% and ear weight by 12.1% and 13.0%, respectively, relative to RS_10-2_.

### 3.6. Kernel Nutritional Quality

**Figure 6:**
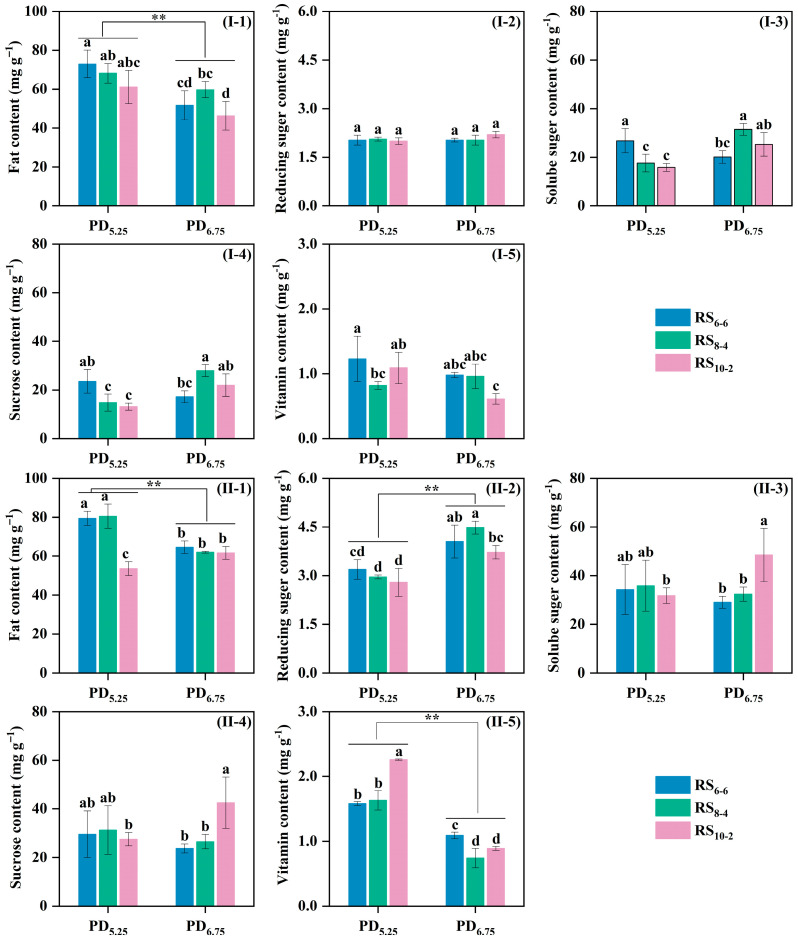
Effects of plant density and row spacing configurations on kernel nutritional quality of waxy maize. (**I-1**,**II-1**) indicate grain fat content data in 2021 and 2022, (**I-2**,**II-2**) indicate grain reducing sugar content data in 2021 and 2022, (**I-3**,**II-3**) indicate grain soluble content data in 2021 and 2022, (**I-4**,**II-4**) indicate grain sucrose content data in 2021 and 2022, (**I-5**,**II-5**) indicate grain vitamin C content data in 2021 and 2022. Different lowercase letters indicate significant differences among treatments at *p* < 0.05. **, significant differences among treatments at *p* < 0.01.

Plant density significantly influenced the kernel fat, reducing sugar and vitamin C in waxy maize, while row spacing significantly affected fat, starch content and vitamin C concentrations (Table 5). Compared to PD_6.75_ treatments, PD_5.25_ treatments increased fat content by 28.2% in 2021 and 13.8% in 2022 (Figure 6I-1,II-1). Under high density (PD_6.75_), reducing sugar content rose 37.0% relative to PD_5.25_ in 2022. Vitamin C content was 1.2% higher in PD_5.25_ than PD_6.75_ in 2022.

## 4. Discussion

### 4.1. Density-Driven Canopy Architecture Modifications

The PH and EH of maize are crucial agronomic traits influencing single-plant yield and density tolerance [27]. Previous studies have indicated that increasing planting density generally leads to taller plants, while EH exhibits a trend of an initial increase followed by a decrease [28]. In the present study, planting density had a negligible impact on overall PH; however, the EH under the PD_6.75_ treatment was significantly greater than that under the PD_5.24_ treatment, concomitant with a significant 2.59% increase in the EHC. This phenomenon is likely associated with the shade avoidance response (SAR) triggered by inter-plant competition under high-density conditions [29]. The SAR involves phytochrome-mediated perception of reduced red to far-red light ratios and subsequent auxin redistribution that promotes internode elongation—a key physiological mechanism underlying canopy architectural adaptation to crowding stress. Within dense canopies, intensified mutual shading deteriorates the light environment in the middle and lower strata. Plants perceive the reduced red to far-red light ratio and initiate the SAR [30], which preferentially promotes the elongation of internodes supporting the ear. This strategic elongation positions the ear at a relatively higher canopy level to capture more light resources [31]. Given the non-significant change in PH observed here, the marked increase in EH inevitably resulted in a significantly higher EHC.

As planting density rises, maize plants typically undergo autonomous adjustments, such as reducing the leaf angle of the three leaves around the ear to create a more erectophile canopy and improve light penetration [32]. Contrary to these findings, the current study demonstrated that both the UL and EL were significantly larger under the PD_6.75_ treatment compared to PD_5.25_. The underlying mechanism for this divergence may involve shade signals modulating auxin transport or distribution at the lamina joint, promoting adaxial-side cell expansion and consequently leading to a larger leaf angle [33]. Alternatively, elevated density may induce the overexpression of the *Zm*DWF1 gene in maize, which has been functionally validated as a positive regulator of leaf angle [31,34]. Moreover, the wide–narrow row configuration (RS_8-4_) was observed to mitigate leaf angle enlargement under high density by improving lateral light penetration, thereby reducing the shade signal intensity that drives leaf angle expansion—demonstrating a clear interaction between planting configuration and morphological response.

To intercept more light energy, the LAI of a maize population typically increases under high planting density [35,36]. Our results align with this expectation, showing that both LAI_R1_ and LAI_R3_ increased significantly with higher density, while the LAD_ER_ and LAD_R_ decreased significantly. This reduction in LAD is potentially attributable to intensified competition for light, water, and nutrients within dense stands, which can lower chlorophyll content, impair photosynthetic capacity [37], and accelerate the senescence of lower leaves [38]. The RS_8-4_ configuration partially alleviated this resource competition by improving light distribution and potentially enhancing water and nutrient uptake through optimized root spatial distribution.

### 4.2. The Density–Yield Paradox: Unveiling the Constraints

Optimizing planting density is a crucial strategy for enhancing maize yield, with efficient dry matter translocation and accumulation serving as the foundational physiological processes [39,40]. Previous research on fresh-eating maize demonstrated that both dry matter accumulation and yield increased with density, specifically when raised from 4.5 × 10^4^ plants ha^−1^ to 6.0 × 10^4^ plants ha^−1^ [41]. However, in the present study, increasing the density from 5.25 × 10^4^ plants ha^−1^ to 6.75 × 10^4^ plants ha^−1^ resulted in a significant increase in both DMA_gm_ and DMA_tm_, yet this was concomitant with a significant reduction in HKW and KNE, ultimately failing to produce a significant yield increase. This outcome diverges from the conclusions reported by Zhang et al. [42], who observed a yield increase with rising density in fresh-eating maize, but aligns with the perspective presented by Ye et al. [43].

The observed discrepancy may be attributed to the fact that under high-density stress, silk elongation rate is slowed and the silking period is delayed, thereby increasing the risk of ovary abortion [44]. Concurrently, excessive density can impair photosynthetic capacity, leading to insufficient photoassimilate production to meet the demands of kernel development [45], which consequently reduces both HKW and KNE. A yield gain can still be realized if the compensatory effect of increased NEE at the population level outweighs the reduction in yield per ear [43]; conversely, if the gain in ear number cannot offset the loss in per-ear yield, overall productivity remains stagnant [46]. Furthermore, compact plant types, characterized by smaller leaf angles and a more ideal canopy structure conferring higher photosynthetic efficiency, are typically more conducive to the allocation of photoassimilates to the ear [47]. However, the cultivar ‘Jinnuo 20’ used in this experiment is semi-compact, and under a high density, both the UL and EL increased significantly, resulting in poorer canopy light penetration. This suboptimal canopy architecture likely limited the population’s photosynthetic capacity and the translocation of dry matter to the grains, thus precluding a yield increase.

### 4.3. The Density Dilemma: Balancing Yield and Quality in Waxy Maize

Rational planting density is conducive to leveraging population effects and coordinating source–sink relationships [48], which is of great significance for improving the market quality of ears and the nutritional quality of kernels in fresh-eating maize. However, as density increases, plants may compete for more resources such as temperature, light, water, and nutrients, leading to a shift in dry matter allocation toward roots, stems, and leaves at the expense of allocation to the ears [49,50]. From both agronomic and commercial perspectives, the reduction in HKW (1000-kernel weight) and deterioration of ear morphology under high density have significant implications: agronomically, HKW reduction indicates impaired grain filling efficiency, while commercially, smaller kernel size and poorer ear appearance directly reduce marketability and consumer appeal of fresh waxy maize products. At the same time, an unreasonable canopy structure under high-density conditions may adversely affect ear development [51,52]. Wang et al. [53] found that when the planting density of fresh-eating maize increased from 4.5 × 10^4^ plants ha^−1^ to 9.9 × 10^4^ plants ha^−1^, both EL and ED showed a significant negative correlation with density, indicating that high density may inhibit morphological development of the ears. Consistent with this, the EL and ED under the PD_6.75_ treatment in this study were significantly lower than those under the PD_5.25_ treatment, resulting in reduced market quality.

Nutritional quality is an important indicator for evaluating the value of fresh-eating maize. Some studies have shown that after increasing planting density, the contents of soluble sugar, sucrose, and starch in grains did not exhibit a decreasing trend [43]. Other studies have reported that reducing sugar content in grains initially increased and then decreased with increasing planting density, while protein, lipid, and starch contents were significantly negatively correlated with density [54]. The results of this study indicate that increased density significantly raised the reducing sugar content in grains, but vitamin C, starch, protein, and fat contents decreased to varying degrees. The reduction in starch content (including waxy starch) is particularly noteworthy both agronomically and commercially, as it directly affects the eating quality and texture of waxy maize—key determinants of consumer preference and market value. This may be related to changes in the allocation of photoassimilates and the activity of key metabolic enzymes. Sucrose, a photosynthetic product, is irreversibly decomposed into reducing sugars after entering the kernel under the catalysis of acid invertase [55]. Increased planting density may upregulate the activity of acid invertase, promoting the accumulation of reducing sugars. On the other hand, high-density stress may also inhibit the activity and gene expression of multiple key synthesis enzymes in the kernels, including ADP-glucose pyrophosphorylase in starch synthesis, acetyl-CoA carboxylase in fatty acid synthesis, glutamine synthetase in nitrogen metabolism, and L-galactose dehydrogenase in vitamin C synthesis. The overall decline in the activity of these enzymes ultimately leads to reduced contents of major nutrients such as starch, fats, proteins, and vitamin C. Furthermore, discrepancies among different studies may be related to variations in climatic conditions, cultivars, and management practices, which require in-depth investigation in future research.

The nutritional quality of fresh-eating maize is typically evaluated based on its rich content of nutrients such as proteins, amino acids, and vitamins [56]. However, excessively increasing planting density can adversely affect its nutritional quality. In summary, increasing planting density generally exhibits negative effects on both the market quality of ears and the nutritional components of kernels in waxy corn, but the underlying physiological and molecular mechanisms require further in-depth study.

### 4.4. Optimizing Row Spacing to Mitigate High-Density Risks in Waxy Maize

A rational row spacing configuration can effectively optimize the canopy structure of maize populations, moderately regulate planting density, and significantly influence yield. This study demonstrated that under the PD_5.25_ treatment, there was no significant yield difference between uniform row spacing and alternating wide–narrow row planting. However, at the PD_6.75_ density, the yield under the RS_8-4_ wide–narrow row treatment was significantly higher than that under the RS_6-6_ treatment, without adversely affecting quality—a result largely consistent with the findings of Dong et al. [57]. This interaction effect can be biologically interpreted through improved light capture efficiency and a more uniform resource distribution: the wide–narrow row configuration creates alternating zones of high and low plant density, reducing inter-plant competition for light, water, and nutrients while maintaining overall planting density—a mechanism that enhances resource use efficiency under high-density stress. Further analysis revealed that compared to the RS_6-6_ treatment, the RS_8-4_ treatment under an increased density improved ear length and ear weight by 5.52% and 10.61%, respectively, while not negatively impacting kernel nutritional content, thereby enhancing certain market traits of the ears. This improvement may be attributed to the RS_8-4_ treatment’s ability to optimize the canopy structure of fresh-eating maize and enhance the light capture capacity within the population [58]. Moreover, the RS_8-4_ treatment increased the SPAD values of the three leaves around the ear [59], promoting photosynthesis [60] and facilitating the efficient translocation and accumulation of photosynthetic assimilates into the kernels. From a practical perspective, these findings suggest that wide–narrow row configurations can significantly improve light and water use efficiency in waxy maize production systems, particularly under the semi-arid conditions of the Loess Plateau where resource limitation is a major production constraint. In conclusion, a rational wide–narrow row configuration can mitigate the risk of yield reduction under high-density planting while maintaining the stability of fresh-eating maize quality, representing a feasible cultivation management strategy.

### 4.5. Limitations and Prospects

Our study was conducted at a single experimental station over two consecutive growing seasons. While this design allowed for controlled comparisons of planting configurations, we recognize that the generalizability of our findings may be constrained by the specific environmental conditions of our study site. To address this limitation, future research will incorporate multi-location trials across different ecological regions of the Loess Plateau, spanning multiple growing seasons to account for inter-annual climatic variability.

Conventional planting density for fresh-eating maize typically ranges around 5.25 × 10^4^ plants ha^−1^. This study employed two density regimes (5.25 × 10^4^ plants ha^−1^ and 6.75 × 10^4^ plants ha^−1^) to examine density-dependent effects on growth dynamics and yield formation. While the optimal density threshold remains indeterminate from current findings, the higher density treatment (6.75 × 10^4^ plants ha^−1^) likely exceeded the supra-optimal threshold, imposing developmental constraints. Conversely, the lower density (5.25 × 10^4^ plants ha^−1^) failed to manifest the anticipated yield advantages associated with moderate density elevation. Subsequent research will incorporate expanded density gradients to systematically elucidate density-mediated regulation of agronomic traits, including photosynthetic efficiency, biomass partitioning, yield architecture, and quality parameters.

Conventional row configurations for fresh-eating maize production typically employ equidistant spacings of 50 cm or 60 cm. To explore density-compatible planting systems, this study implemented contrasting row configurations: 60-cm equidistant (60:60), 80-cm/40-cm dual (80∶40), and 100-cm/20-cm dual (100:20) spacings. Notably, most agronomic traits exhibited non-significant responses to row configuration treatments. This apparent insensitivity may indicate that the 60-cm equidistant spacing provides optimal resource capture efficiency, facilitating superior utilization of radiation and thermal resources. Subsequent research will incorporate expanded spacing gradients—including sub-50-cm configurations—to systematically elucidate the effects of row geometry on canopy architecture, photoassimilate partitioning, yield components, and quality attributes.

The experimental material comprised Jinnuo 20, a black-pericarp waxy maize cultivar developed by Shanxi Agricultural University in 2019. This cultivar is listed among the provincially promoted fresh-eating maize varieties certified by the Shanxi Department of Agriculture and Rural Affairs. Nevertheless, the current dominance of diversified commercial cultivars, such as Wannuo 2000, Jingkenuo 2000, Caitiannuo 1958, and Heitiannuo 631, imposes constraints on extrapolating findings derived from a single genotype. Subsequent research will therefore incorporate elite cultivars with extensive commercial adoption to establish genotype-specific management practices for synergistic enhancement of yield potential and resource use efficiency.

## 5. Conclusions

The RS_8-4_ dual-row configuration demonstrated the most pronounced positive effects on morphological development, dry matter partitioning, and yield formation. Although increasing planting density reduced kernel nutritional quality parameters—notably vitamin C and starch content—within the tested density range, it exerted negligible influence on reducing sugars, sucrose, and total soluble sugars. Critically, the RS_8-4_ wide–narrow row configuration represents a practicable cultivation strategy that simultaneously achieves both yield stability and quality preservation in high-density ‘Jinnuo 20’ production systems on the Loess Plateau. This approach may also serve as a valuable reference for the cultivation of other regional varieties in similar agroecological conditions.

## Figures and Tables

**Table 1:** Analysis of variance for plant morphological traits, leaf angle, LAI and LAD.

ANOVA	PH	SD	EH	EHC	UL	EL	LL	LAI_R1_	LAI_FH_	LAD_ER_	LAD_FH_
PD	ns	ns	*	**	**	**	ns	**	**	**	**
RS	ns	ns	ns	ns	ns	ns	ns	ns	ns	ns	ns
Y	**	ns	–	–	–	–	–	ns	**	**	**
PD × RS	ns	ns	**	**	**	**	ns	ns	ns	ns	ns
PD × Y	ns	**	–	–	–	–	–	ns	ns	ns	ns
RS × Y	ns	ns	–	–	–	–	–	ns	ns	ns	ns
PD × RS × Y	ns	ns	–	–	–	–	–	*	ns	*	ns

PH, plant height. SD, stem diameter. EH, ear height. EHC, ear height coefficient. UL, insertion angle of upper ear leaf. EL, insertion angle of primary ear leaf. LL, insertion angle of lower ear leaf. LAI_R1_, leaf area index at the silking stage. LAI_FM_, leaf area index at fresh-market maturity. LAD_ER_, leaf area duration from emergence to silking. LAD_FM_, leaf area duration from silking to fresh-market maturity. * and **, significant at the 0.05 and 0.01 levels, respectively; ns, no significant; –, data not available.

**Table 2:** Dry matter accumulation and translocation in waxy maize at silking and fresh-market maturity stages during the 2022 growing season.

Plant Density	Row Spacing	The Silking Stage			Fresh-Market Maturity				DMT (t ha^−1^)	DMTE(%)	CDMG(%)	HI
		DMA_vos_ (t ha^−1^)	DMA_es_ (t ha^−1^)	DMA_ts_ (t ha^−1^)	DMA_vom_ (t ha^−1^)	DMA_bcm_ (t ha^−1^)	DMA_gm_ (t ha^−1^)	DMA_tm_ (t ha^−1^)				
PD_5.25_	RS_6-6_	6.78 ± 0.73 a	2.99 ± 0.08 a	9.77 ± 0.66 ab	4.90 ± 0.38 c	1.64 ± 0.24 ab	2.49 ± 0.48 ab	9.03 ± 0.69 b	3.24 ± 1.40 a	32.58 ± 11.70 a	130.19 ± 43.50 a	0.28 ± 0.06 a
	RS_8-4_	7.01 ± 0.37 a	3.15 ± 0.97 a	10.16 ± 1.02 ab	5.94 ± 1.17 bc	1.99 ± 0.35 ab	2.17 ± 0.77 ab	10.10 ± 1.34 b	2.22 ± 2.60 a	20.23 ± 22.30 a	166.79 ± 243.90 a	0.21 ± 0.05 ab
	RS_10-2_	7.10 ± 0.77 a	2.21 ± 0.90 a	9.31 ± 1.58 ab	6.64 ± 0.52 ab	1.66 ± 0.18 ab	1.09 ± 0.26 b	9.39 ± 0.40 b	1.00 ± 1.50 a	8.89 ± 13.90 a	68.26 ± 125.10 a	0.11 ± 0.02 b
PD_6.75_	RS_6-6_	8.05 ± 0.18 a	3.17 ± 0.37 a	11.22 ± 0.51 a	5.48 ± 0.27 bc	2.18 ± 0.48 ab	2.77 ± 0.63 a	10.43 ± 1.68 b	3.55 ± 1.50 a	31.23 ± 11.70 a	137.80 ± 66.70 a	0.26 ± 0.05 a
	RS_8-4_	7.28 ± 0.83 a	2.56 ± 0.65 a	9.84 ± 1.05 ab	7.62 ± 0.39 a	2.44 ± 0.37 a	3.50 ± 1.02 a	13.56 ± 2.25 a	0.74 ± 0.20 a	7.15 ± 2.20 a	23.82 ± 15.70 a	0.25 ± 0.05 a
	RS_10-2_	6.87 ± 0.48 a	1.86 ± 0.43 a	8.73 ± 0.74 b	5.58 ± 0.10 bc	1.55 ± 0.15 b	2.60 ± 0.31 a	9.73 ± 0.15 b	1.62 ± 1.00 a	17.85 ± 9.80 a	60.08 ± 31.30 a	0.27 ± 0.02 a
ANOVA												
PD		ns	ns	ns	ns	ns	*	*	ns	ns	ns	*
RS		ns	ns	ns	**	ns	ns	*	ns	ns	ns	ns
PD × RS		ns	ns	ns	*	ns	ns	ns	ns	ns	ns	ns

DMA_vos_, DMA_es_ and DMA_ts_ indicate dry matter accumulation in vegetative organs, ears and total plants (excluded root) at silking stage, respectively. DMA_vom_, DMA_bcm_, DMA_gm_ and DMA_tm_ indicate dry matter accumulation in vegetative organs, bract + cob, grains and total plant (excluded root) at fresh-market maturity stage, respectively. Vegetative organs include the leaf, stem and sheath. Different lowercase letters within a column indicate significant differences among treatments at *p* < 0.05. *, ** significant at *p* < 0.05 and *p* < 0.01, respectively; ns, not significant.

**Table 3:** Yield components of waxy maize under different plant densities and row spacing configurations.

Year	Plant Density	Row Spacing	NEE (10^4^ Ears ha^−1^)	KNE	HKW (g)	ThY (t ha^−1^)	FEY (t ha^−1^)
2021	PD_5.25_	RS_6-6_	4.67 ± 0.37 c	698.67 ± 68.17 a	27.90 ± 3.41 a	9.03 ± 1.01 a	12.16 ± 0.58 a
		RS_8-4_	4.22 ± 1.00 d	689.73 ± 68.91 ab	26.30 ± 3.78 ab	7.66 ± 2.24 b	11.17 ± 3.19 a
		RS_10-2_	4.61 ± 0.21 c	702.80 ± 94.03 a	26.60 ± 2.95 a	8.61 ± 1.41 ab	11.39 ± 0.19 a
	PD_6.75_	RS_6-6_	5.61 ± 0.49 a	615.40 ± 50.65 d	24.81 ± 3.35 bc	8.52 ± 1.22 ab	10.67 ± 1.64 a
		RS_8-4_	5.22 ± 0.56 b	657.07 ± 79.58 bc	23.21 ± 8.15 c	7.91 ± 2.89 ab	11.06 ± 2.24 a
		RS_10-2_	5.28 ± 0.44 b	646.00 ± 72.05 cd	23.40 ± 5.17 c	8.05 ± 2.33 ab	11.33 ± 1.69 a
2022	PD_5.25_	RS_6-6_	3.78 ± 0.44 b	702.53 ± 80.89 a	28.19 ± 3.83 a	7.56 ± 1.90 a	10.06 ± 0.59 a
		RS_8-4_	3.00 ± 1.00 c	661.60 ± 67.44 b	25.47 ± 4.81 b	5.30 ± 2.43 de	8.00 ± 3.68 a
		RS_10-2_	2.67 ± 0.14 c	673.47 ± 60.01 ab	25.69 ± 2.68 b	4.61 ± 0.67 e	6.72 ± 0.69 a
	PD_6.75_	RS_6-6_	3.89 ± 0.76 b	644.07 ± 75.37 b	25.21 ± 2.51 b	6.32 ± 1.61 bc	9.06 ± 1.83 a
		RS_8-4_	4.28 ± 0.98 a	665.87 ± 63.37 ab	25.02 ± 2.89 b	7.19 ± 2.15 ab	9.94 ± 2.50 a
		RS_10-2_	4.11 ± 0.08 ab	601.53 ± 78.94 c	24.14 ± 4.30 b	6.00 ± 1.51 cd	8.89 ± 0.92 a
ANOVA							
PD			**	**	**	ns	ns
RS			ns	ns	ns	ns	ns
Y			**	ns	ns	ns	**
PD × RS			ns	*	ns	ns	ns
PD × Y			ns	*	ns	ns	ns
RS × Y			ns	*	ns	ns	ns
PD × RS × Y			ns	ns	ns	ns	ns

NEE, number of effective ears. KNE, kernel number per ear. HKW, 100-kernel weight. ThY, the theoretical yield. FEY, fresh ear yield. Different lowercase letters following mean values indicate significant differences among treatments at *p* < 0.05. * and **, significant differences among treatments at *p* < 0.05 and *p* < 0.01, respectively. ns, not significant.

**Table 4:** Effect of plant densities and row spacing configurations on ear traits of waxy maize.

Year	Planting Density	Row Spacing	Ear Length (cm)	Ear Weight (g)	Ear Diameter (mm)
2021	PD_5.25_	RS_6-6_	19.5 ± 2.1 a	260.1 ± 27.4 a	46.9 ± 2.0 ab
		RS_8-4_	18.2 ± 2.0 b	248.6 ± 29.6 a	46.9 ± 2.3 ab
		RS_10-2_	18.8 ± 1.4 ab	249.4 ± 26.8 a	48.1 ± 2.0 a
	PD_6.75_	RS_6-6_	16.3 ± 1.8 e	206.4 ± 32.7 c	45.9 ± 2.0 b
		RS_8-4_	17.2 ± 1.7 c	228.3 ± 28.0 b	45.9 ± 5.8 b
		RS_10-2_	16.8 ± 1.4 cd	208.7 ± 25.5 c	45.7 ± 1.8 b
2022	PD_5.25_	RS_6-6_	20.3 ± 2.2 a	285.1 ± 34.4 a	47.7 ± 2.0 a
		RS_8-4_	19.7 + 1.2 ab	265.7 ± 30.9 b	45.1 ± 2.4 bc
		RS_10-2_	19.2 ± 1.0 bc	263.1 ± 23.0 bc	46.0 ± 1.8 b
	PD_6.75_	RS_6-6_	18.8 ± 1.2 c	248.2 ± 24.6 c	45.7 ± 1.6 bc
		RS_8-4_	18.8 ± 1.4 c	250.2 ± 20.0 bc	45.7 ± 1.8 bc
		RS_10-2_	17.0 ± 1.7 d	221.4 ± 40.4 d	44.8 ± 2.1 c
ANOVA					
PD			**	**	**
RS			**	*	**
Y			**	ns	**
PD × RS			**	**	ns
PD × Y			ns	*	ns
RS × Y			**	ns	**
PD × RS × Y			*	ns	ns

Different lowercase letters indicate significant difference at *p* < 0.05. * and **, significant differences among treatments at *p* < 0.05 and *p* < 0.01, respectively. ns, not significant.

**Table 5:** Analysis of variance for kernel nutritional quality traits in waxy maize.

	Fat Content	Reducing Sugar Content	Soluble Content	Sucrose Content	Vitamin C Content
PD	**	**	ns	ns	**
RS	**	ns	ns	ns	**
Y	**	**	**	**	**
PD × RS	**	ns	*	**	**
PD × Y	ns	**	ns	ns	**
RS × Y	ns	*	ns	*	**
PD × RS × Y	**	ns	ns	*	**

* and **, significant differences among treatments at *p* < 0.05 and *p* < 0.01, respectively. ns, not significant.

## Data Availability

Data are contained within the article.
